# Neuraxial Anesthesia in the Geriatric Patient

**DOI:** 10.3389/fmed.2018.00254

**Published:** 2018-09-24

**Authors:** Atanas G. Sivevski, Dafina Karadjova, Emilija Ivanov, Andrijan Kartalov

**Affiliations:** ^1^Department of Anaesthesia, Clinical Center Mother Teresa, University Clinic for Gynecology and Obstetrics, Skopje, Macedonia; ^2^Clinical Center Mother Teresa, University Clinic for TOARILUC, Skopje, Macedonia

**Keywords:** geriatric (aging), neuraxial anesthesia, low-dose, opoids, hypotension

## Abstract

Neuraxial anesthesia is recommended as a well-accepted option to minimize the perioperative side effects in the geriatric patients. The available data from the current researches have shifted the focus from the conventional approach to spinal anesthesia to the concept of low dose local anesthetic combined with opioids. What remains clear from all these studies is that hemodynamic stability is much better in patients who received low-doses of intrathecal bupivacaine in combination with opioids, which is possibly result of a potent synergistic nociceptive analgesic effect and their minimal potential effects on sympathetic pathways thus minimizing spinal hypotension. Spinal anesthesia with 5–10 mg of 0.5% heavy bupivacaine, fentanyl 20 mcg and 100 mcg of long-acting morphine added to the perioperative plan decreased the incidence of spinal hypotension and improved perioperative outcomes in the geriatric patients undergoing (low segment) surgical procedures. These findings may be of interest in the gynecologic geriatric surgery also in which area there are very few studies concerning the use of low-dose concept.

## Introduction

The European population is growing older. It is anticipated that in the next years more than 30% will be older than 65. Particularly fast growing sub-population is the one older than 85 (1). The anatomical and physiological changes of aging present challenge for the anesthetic and surgical management.

There is no ideal anesthetic for geriatric patients. More important than the choice of anesthetic technique is adequate pre-operative assessment and planning of appropriate monitoring. Together with tight control of the perioperative physiological parameters it will favor positive patient outcomes. The decrease of hospital stay in elderly patients undergoing surgery decreases the incidence of adverse events like reduction of both respiratory events and nosocomial infections, as well a less postoperative cognitive dysfunction (POCD) at 1 week in the postoperative period.

The physiological changes of aging have impact on neuraxial anesthesia techniques, both spinal and epidural. With advancing age, as a result of some anatomical irregularities (reduction in number of neurons, deterioration of myelin sheaths, sclerotic closure of the intervertebral foramens etc.) the level of analgesia increases after epidural administration of local anesthetics (LA) (2). With spinal anesthesia, the level of analgesia also increases after spinal administration of hyperbaric solutions (3).

## Clinical researches

There is no controlled trial that can demonstrate that either neuraxial or general anesthesia is clearly superior in terms of outcome in elderly patients. Cochrane systematic review of studies of hip surgeries in elderly, looked at 17 trials (2,567 patients) comparing general to neuraxial anesthesia. The review concluded that the long-term mortality was equal for both groups (4). However, neuraxial anesthesia remains a well-accepted option to: minimize the surgical stress (tachycardia and hypertension), reduce the pulmonary compromise (atelectasis, pneumonia, prolonged mechanical ventilation), thus showing superior postoperative pain control and reduction of perioperative opioids consumption, hence minimizing opioid side effects. Neuraxial anesthesia results in better peripheral vascular circulation and reduces total blood loss. Neuraxial anesthesia also reduces the incidence of POCD in the first postoperative week (4). In addition, neuraxial anesthesia favors early ambulation thus preventing deep venous thrombosis.

Results from the current research have shifted the focus from the conventional approach to spinal anesthesia (SA) to the concept of low dose LA combined with lipophilic opioids. The antinociceptive synergism between LA and intrathecal (IT) opioids is well known. This concept provides effective and superior analgesia and may prolong the duration and effectiveness of postoperative analgesia without associated motor blockade (5, 6). Most importantly, use of low dose of LA reduces the incidence of hemodynamic side effects in elderly (7). Carpenter at al. identified the high levels of sensory anesthesia and increasing age as two main risk factors for the development of spinal hypotension (8). In elderly, SA is associated with 25–69% incidence of hypotension (8). Decreased physiologic reserve and increased incidence of systemic disease, particularly cardiovascular disease, make the elderly population prone to long term complications even with brief episodes of uncorrected hypotension (9, 10).

Fentanyl is the most frequently used intrathecal lipophilic opioid and when administered in single dose of 10–30 mcg, it has a rapid onset (10–20 min) and short duration of action (4–6 h) with minimal cephalic spread. These properties minimize the risk of delayed respiratory depression and favor use of intrathecal fentanyl in ambulatory anesthesia where enhanced analgesia without prolonged hospital stay is important (11, 12).

Morphine is unsuitable for ambulatory surgery because of its slow onset time (30–60 min), dose-related duration of analgesia (13–33 h) and side-effect profile, particularly the delayed onset respiratory depression. Administration of up to 200 mcg of IT morphine with LA for peripheral vascular surgery in elderly patients (average age of 68) can be safely performed with minimal adverse respiratory risk (9). Two meta-analyses determined the incidence of respiratory depression in patients receiving low-dose (<0.2 and 0.2–0.3 mg) IT morphine to be 0–1.2% (13, 14). A retrospective audit of IT morphine in adult patients (409) where more than half the patients (57.2%) were aged 70 years or older and the doses of IT morphine used ranged from 0.1, 0.15, or 0.2 mg (22.9, 30.2, and 45.3%) only one patient, a 74 old-male with past history of cerebrovascular accident and epilepsy, developed respiratory depression (0.24%) (15).

Other researchers concluded that 100 mcg of morphine added to the spinal anesthetic (hip surgery) provided the most optimal balance between analgesia, pain relief and pruritus with acceptable risk of respiratory depression (16). There was no case of respiratory depression (Sat < 94%, RR < 8) or sedation score>2 in all of these studies. Therefore, we consider the risk of respiratory depression with 100 mcg of IT morphine added to low-dose mixture of LA plus fentanyl to be minimal and patients can leave the hospital the day after surgery. Also, the 100 mcg IT morphine could provide post-operative analgesia for the first 24 h, which is superior to the short-term fentanyl-analgesia in the immediate post-operative period.

Other studies with low dose bupivacaine and sufentanil showed similar results (17). Kumar et al reported hypotension in 44% in the conventional group (12.5 mg of 0.5% hyperbaric bupivacaine in a total 2.5 ml), and only 8% in the low-dose group, while Olofsson et al. treated 88% patients for hypotension in their conventional SA group (18, 19). The higher incidence of hypotension in these studies might be the different definition of hypotension. What remains clear from all these studies is that hemodynamic stability is much better in patients who received low-doses of intrathecal bupivacaine in combination with opioids, which is possibly result of a potent synergistic nociceptive analgesic effect and their minimal potential effects on sympathetic pathways thus minimizing spinal hypotension.

Besides lower incidence of hypotension, low-dose SA is characterized with less intense and shorter motor block, although the degree of motor block was not so important for this type of surgical procedure (17, 19). Nevertheless, the time in bed and ambulation was decreased, consequently reducing the incidence of potential postoperative complications. The post-operative pain was also at a satisfactory low level, while the percentage of side-effects minimal. Similar observations were found in other similar studies in the geriatric population (20, 21).

Elderly patients undergoing neuraxial anesthesia are at increased risk of hypothermia and shivering, which can lead to increased oxygen consumption, ventilation and cardiac output. There are studies showing that addition of fentanyl to low-dose bupivacaine decrease the incidence of shivering during spinal anesthesia in elderly patients (12, 18).

There are more options for adding adjuvants to LA in the SA. However, the opioids and α_2_ adrenergic agonists are more commonly used as adjuvants in clinical practice. Dexmedetomidine, a selective α_2_ adrenergic receptor agonist, has been shown to be a better adjuvant of LAs for neuraxial blocks although clonidine is the first clinically used intrathecal α_2_-adrenoreceptor agonist.

Adding dexmedetomidine to LA in the SA is a relatively new area and there are almost no data on its use in an elderly population. In a systemic review and meta-analytic study dated from 2017, where researchers independently searched the PUBMED, EMBASE, Cochrane library and CBM for randomized controlled trials comparing the effects of dexmedetomidine and fentanyl as adjuvants to LAs for intrathecal injection (total of 639 patients from 9 studies), was reported that compared to fentanyl, dexmedetomidine as LA adjuvant in spinal anesthesia prolonged the duration of spinal anesthesia, improved postoperative analgesia, reduced the incidence of pruritus, and did not increase the incidence of hypotension and bradycardia (22).

There is no relevant evident data when comparing a dexmedetomidine with fentanyl-morphine combination as an adjuvant to LA in SA and especially applied in the elderly patients.

## Conclusion

Current clinical evidence-based recommendations conclude that neuraxial opioid procedure must be one of the most important skills to master for the treatment of perioperative pain in the geriatric patients. This finding involves a broad type of surgical procedures that should benefit from the practice, ranging from minor (ambulatory) surgery to major procedures (23). Neuraxial anesthesia minimizes the risk of common postoperative side-effects seen with general anesthesia including POCD, fatigue, dizziness, pain, and gastrointestinal dysfunction while neuraxial opioids are safer and preferable to parenteral opioids.

A low-dose concept of SA, that consists of LA plus opioids, improved perioperative outcomes in the geriatric patients undergoing elective (low segment) surgery. A combination of intrathecal lipophilic and hydrophilic opioids, such as fentanyl and morphine added to a low dose LA, can improve and prolong perioperative analgesia in the first 24 h postoperatively. It is a simple and practical technique that decreases incidence of spinal hypotension, particularly undesirable in the elderly. The risk of respiratory depression is negligible when used in such a low dose settings. The SA with 5–10 mg 0.5% heavy bupivacaine mixed with fentanyl 20 mcg and morphine 100 mcg could be an acceptable choice in the growing segment of geriatric gynecological population also. There are very few studies concerning neuraxial low-dosage mixture in the geriatric gynecological surgery and more research must be done to confirm safety and efficacy of this concept.

## Author contributions

AS, DK, EI, and AK declare that: (1) they made substantial contributions to the conception or design of the work; (2) they drafted the work and revised it; (3) they approve its publication; and (4) they agree to be held accountable for all aspects of the work in ensuring that questions related to the accuracy or integrity of any part of the work are appropriately investigated and resolved.

### Conflict of interest statement

The authors declare that the research was conducted in the absence of any commercial or financial relationships that could be construed as a potential conflict of interest.
